# ARMNet: A Network for Image Dimensional Emotion Prediction Based on Affective Region Extraction and Multi-Channel Fusion

**DOI:** 10.3390/s24217099

**Published:** 2024-11-04

**Authors:** Jingjing Zhang, Jiaying Sun, Chunxiao Wang, Zui Tao, Fuxiao Zhang

**Affiliations:** 1 Key Laboratory of Acoustic Visual Technology and Intelligent Control System, Ministry of Culture and Tourism, Communication University of China, Beijing 100024, China; an123xinying@cuc.edu.cn (J.S.); cunxiao_cuc@cuc.edu.cn (C.W.); taozui19981214@163.com (Z.T.); fuxiao@cuc.edu.cn (F.Z.); 2 School of Computer and Cyber Sciences, Communication University of China, Beijing 100024, China; 3 Beijing Key Laboratory of Modern Entertainment Technology, Communication University of China, Beijing 100024, China; 4 Center for Ethnic and Folk Literature and Art Development, Ministry of Culture and Tourism, Beijing 100007, China

**Keywords:** image emotion prediction, dimensional emotion space, attention mechanism, eye fixation detection, multi-channel fusion

## Abstract

Compared with discrete emotion space, image emotion analysis based on dimensional emotion space can more accurately represent fine-grained emotion. Meanwhile, this high-precision representation of emotion requires dimensional emotion prediction methods to sense and capture emotional information in images as accurately and richly as possible. However, the existing methods mainly focus on emotion recognition by extracting the emotional regions where salient objects are located while ignoring the joint influence of objects and background on emotion. Furthermore, in the existing literature, when fusing multi-level features, no consideration has been given to the varying contributions of features from different levels to emotional analysis, which makes it difficult to distinguish valuable and useless features and cannot improve the utilization of effective features. This paper proposes an image emotion prediction network named ARMNet. In ARMNet, a unified affective region extraction method that integrates eye fixation detection and attention detection is proposed to enhance the combined influence of objects and backgrounds. Additionally, the multi-level features are fused with the consideration of their different contributions through an improved channel attention mechanism. In comparison to the existing methods, experiments conducted on the CGnA10766 dataset demonstrate that the performance of valence and arousal, as measured by Mean Squared Error (MSE), Mean Absolute Error (MAE), and Coefficient of Determination (R²), has improved by 4.74%, 3.53%, 3.62%, 1.93%, 6.29%, and 7.23%, respectively. Furthermore, the interpretability of the network is enhanced through the visualization of attention weights corresponding to emotional regions within the images.

## 1. Introduction

Emotion analysis is a critical research area that aims to enhance human–computer interaction and enable intelligent sensing of emotions [1,2,3]. Effective emotion sensing involves accurately perceiving and interpreting the subtle emotional states depicted in images. In mainstream research, image emotion prediction is usually performed based on dominant affective categories, which include six or eight basic emotion categories [4,5]. However, discrete affective spaces can only represent basic affective categories and are insufficient for subtle emotion representation. With the increasing demand for subtle emotional sensing and representation, image emotion analysis based on dimensional emotion space gradually attracts extensive attention from researchers.

In contrast, dimensional emotion spaces use precise numerical values to represent emotions, making them better suited for describing subtle emotional details [6,7], such as the Pleasure–Activation–Dominance (PAD) model and the valence–arousal–dominance (VAD) model. In this way, dimensional emotion spaces provide an infinite range of emotions and preserve intermediate emotion states, offering a more comprehensive emotional sensing capability [8]. Therefore, dimensional emotion prediction has broader applications in multimedia and other fields, such as intelligent advertising, multimedia retrieval, and public opinion analysis.

Currently, most of the public dimensional emotion annotated image datasets, such as International Affective Picture System (IAPS) [9], Nencki Affective Picture System (NAPS) [10], Geneva Affective Picture Database (GAPED) [11], and Open Affective Standardized Image Set (OASIS) [12], are built based on 2D or 3D emotion models. These datasets are small, generally containing around 1000 images per dataset. In 2017, Kim et al. established the first large-scale dataset named CGnA10766 [13], which included 10,766 images based on dimensional emotion annotations. When the dataset contains a considerable number of samples, deep-learning techniques can be used to solve complex problems [13,14,15]. In addition, Kim et al. proposed the first deep learning-based prediction model combining the different levels of features and proved that these features are related to emotions [13]. In 2019, Zhao et al. developed a polarity-consistent deep attention network (PDANet) that integrates spatial and channel-wise attention into a convolutional neural network (CNN) with an emotion polarity constraint. Experiment results demonstrated that the PDANet outperforms the state-of-the-art approaches [16]. In 2021, Li et al. proposed a spatial and channel-wise attention-based emotion prediction (SCEP) model that uses the results from saliency detection for spatial attention and leverages spatial and channel-wise attention, multi-layer characteristics [17]. Alarcão et al. extracted 30 discriminant handcrafted features and analyzed the impact of using the discriminant handcrafted features on three well-known CNNs to identify the feature’s contribution [8].

Dimensional emotion spaces use precise numerical values to represent emotions. This high-precision representation of emotion requires dimensional emotion prediction methods to capture emotional information in images as accurately and richly as possible. However, existing methods mainly extract affective regions with the salient objects, which starts from the object level [18,19], but they only focus on a few affective regions with the salient objects, neglecting the joint influence of objects and background. Most image emotion research fuses multi-level features through skip connections [20,21], which means that these methods do not take into account the differences between the low-level features and the high-level features [22]. Therefore, they fused multi-level features without considering their different contributions to emotion prediction, which makes it challenging to discriminate between valuable and useless features and cannot enhance the utilization of effective features.

An image prediction network based on dimensional emotion, joint affective region, and multi-channel fusion (ARMNet) is designed to solve the above problems. The main contributions of this paper are as follows:

(1) A method for extracting union affective regions, combining eye fixation detection and attention detection, is proposed to expand the effective emotional area. This method can extract the joint affective regions composed of the objects and the background, which has high contributions to emotion prediction.
(2) An improved channel attention mechanism is proposed, which increases the gating mechanism and fuses the multi-level features to consider the different contributions from multi-level features through attention-based weight adaptive adjustment.

## 2. Related Work

### 2.1. Image Emotion Analysis Based on Specific Affective Regions

The key to image emotion analysis is to extract the appropriate discriminant features [16] and those specific affective regions that have a strong influence when triggering emotion [18,23]. Based on the definition of local regions, there are two main methods that exist to extract the specific affective regions. One of the methods extracts specific affective regions based on segmentation or object detection. Xiong et al. segmented local sentiment regions by considering the similarities of colors and textures [24]. Yang et al. used the EdgeBoxes tool to generate thousands of candidate regions and then selected the affective regions automatically by calculating the emotion and object scores of each candidate region [25]. Rao et al. used Faster-RCNN to generate candidate boxes with emotions instead of objects [20]. Another method extracts specific affective regions using the spatial attention mechanism. She et al. used a cross-spatial pooling strategy in the detection branch to generate the spatial attention weight map [23]. Yao et al. extracted polarity- and emotion-specific attended representations by utilizing polarity-specific attention and specific affective attention in lower and higher layers, respectively [26]. Zhao et al. proposed integrating spatial and channel attention into CNN so that both spatial and channel attention could be considered [16]. Li et al. took the results of the salient object detection as an attention distribution to pay attention to every feature entry of multi-layer feature maps [17]. They suppressed the irrelevant regions via a progressive attention process over multiple layers.

Likewise, to highlight the degree of human attention to the information-rich regions, more methods have been proposed using saliency object detection to locate the emotional areas of images [18,19]. The eye fixation detection module can also predict the likelihood of where the human eyes are staring. Wang et al. quantified the performance of human eye detection networks based on deep learning [27]. The results showed that the Salicon [28] has relatively superior comprehensive performance. However, when the number of salient regions in an image is relatively small, current methodologies often neglect a substantial amount of non-salient emotional information.

### 2.2. Image Emotion Analysis Based on Multi-Level Features Fusion

Some studies tried to model this phenomenon using multi-level image features to predict emotion [20,22,29]. For example, Rao et al. [20] proposed a multi-level depth representation network (MldrNet) with a backbone and four branches, which fused the multi-level depth representation with the Mean function. Zhu et al. proposed a BI-GRU framework for visual emotion recognition based on the assumption that features are at different levels [21]. Nagappan et al. presented a multi-stream feature extraction method that captures object and scene features for emotion prediction, integrating various deep image features using a multi-layer perceptron (MLP) and multi-task learning [30]. Rapolu et al. introduced a deep convolutional neural network (CNN) fusion technique that utilizes a differential CNN for extracting emotional features and a supplementary CNN for capturing central object details, significantly enhancing image emotion recognition performance compared to existing state-of-the-art methods [31]. However, most methods fused multi-level features by simply concatenating multi-level features into one. This simplistic fusion approach, characterized by a straightforward skipping connection, overlooks the distinct contributions of high-level and low-level features. However, these features’ roles in emotion prediction are not equivalent; thus, enhancing the multi-layer feature fusion method is essential.

## 3. Method

**Figure 1:**
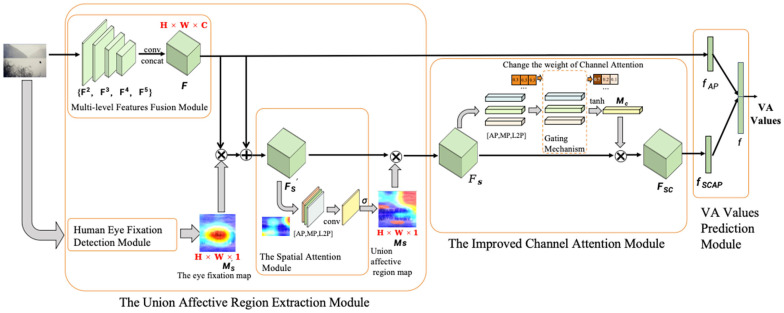
Illustration of the ARMNet.

In this paper, an image-dimension emotion prediction network, joint affective region, and multi-channel fusion named ARMNet is designed to predict the valence and arousal (VA) values of images, as shown in Figure 1. The ARMNet comprises three components: the union affective region extraction module, the improved channel attention module, and the VA values prediction module. Firstly, the union affective region extraction module utilized a pre-trained ResNet-101 [32] to extract image features, followed by the combined detection of eye fixation and spatial attention to generate the union affective regions (details in Section 3.1). Secondly, the improved channel attention module fuses the multi-level features by considering their different contributions through attention-based weight adjustment (details in Section 3.2). Finally, the ARMNet predicts the emotion of the image based on the valence–arousal space through three fully connected layers (details in Section 3.3). The training of the entire framework is performed in an end-to-end manner.

### 3.1. The Union Affective Region Extraction Module

#### 3.1.1. The Multi-Level Features Fusion Module

**Figure 2:**
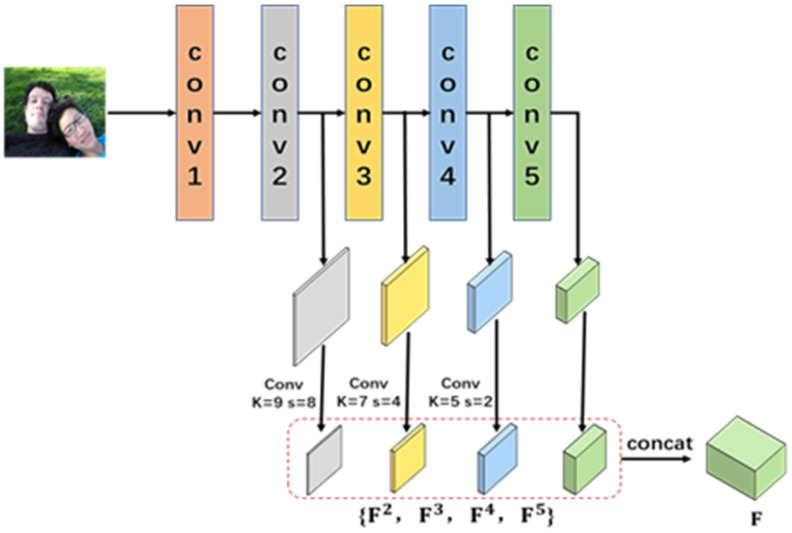
The multi-level features fusion module.

To extract the image features, the pre-trained ResNet101 is selected as the backbone of the multi-level features fusion module. The features of Conv2, Conv3, Conv4, and Conv5 branches are defined as {$F^{2}$, $F^{3}$, $F^{4}$, $F^{5}$}, as shown in Equation (1). The Conv1 is not included due to its large memory cost.

(1) $$F^{l}=\left[f_{1}^{c^{l}},f_{2}^{c^{l}},\ldots .,f_{x^{2}}^{c^{l}}\right]\in R^{c^{l}x^{2}}$$

where $c^{l}\;$ is the number of channels, l is the number of branches, and x is the length of the feature. After the dimension in convolution mode is decreased, the spatial size of features is identical to $x^{2}$. {$F^{2}$, $F^{3}$, $F^{4}$, $F^{5}$} are concatenated along the channel dimension to obtain the multi-level feature F, as shown in Equation (2) and illustrated in Figure 2.

(2) $$F\in R^{\left(c^{2}+c^{3}+c^{4}+c^{5}\right)x^{2}}$$

#### 3.1.2. The Human Eye Fixation Detection Module

To generate the union effective region, the Salicon [28] network is directly integrated into the eye fixation detection module. Then, the raw image is injected into the pre-trained Salicon network to obtain the eye fixation map $M_{S}^{'}$.

#### 3.1.3. The Spatial Attention Module

The spatial attention module can attend to a significant amount of non-salient emotional regions to generate the union affective region. In the Convolutional Block Attention Module (CBAM), the spatial attention module adopts both AvgPool and MaxPool to improve the scale of the feature set [33]. But, AvgPool and MaxPool cannot capture the details of spatial information, which leads to the problem that CBAM has insufficient information when guiding attention learning. Sermanet et al. [34] proposed the concept of L2Pool and claimed that its generalization ability is better than MaxPool. The calculation process is shown in Equation (3).

(3) $$L2Pool\left(f_{h\times w}\right)={\left(\frac{1}{c}\sum_{i=1}^{c}f_{i}{\,}^{2}\right)}^{\frac{1}{2}}$$

**Figure 3:**
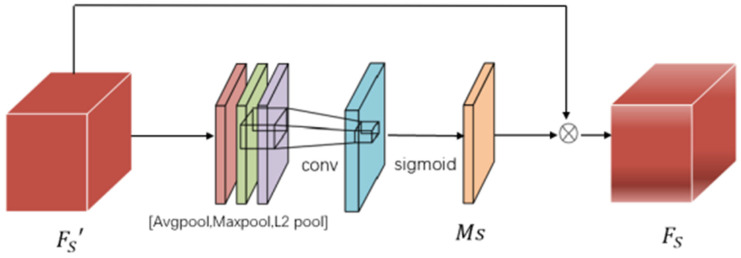
Spatial attention module.

To enrich the feature description and extract useful intermediate features, L2Pool was added to calculate the two-dimensional spatial information as an additional feature descriptor of CBAM, as shown in Figure 3 and Equation (4).

(4) $$F_{S}=M_{S}⊗F_{S}{\,}^{'}$$

For the ARMNet, after the eye fixation detection module processes the image, the multi-layer feature is weighted by the eye fixation map to obtain saliency information, as shown in Equation (5).

(5) $$F_{S}{\,}^{'}=\left(M_{S}^{'}+1\right)⊙F$$

$F_{S}{\,}^{'}$ is fed into the attentional module to generate the spatial attention map and the channel attention map. First, $F_{S}{\,}^{'}$ is received by the spatial attention module. Then, three context descriptions, $AvgPool\left(F_{S}{\,}^{'}\right)$, $MaxPool\left(F_{S}{\,}^{'}\right)$, and $L2Pool\left(F_{S}{\,}^{'}\right)$, were derived using  AvgPool, MaxPool and L2Pool operations, respectively. And these Spatial context descriptors are concatenated along the channel dimension noted as $\hat{F_{S}^{'}}$. Then, the union affective region map $M_{S}\in R^{H\times W\times 1}$ is generated via a convolution layer and a Sigmoid activation function successively, as shown in Equation (6).

(6) $$Ms=\sigma \left(conv\left(\hat{F_{S}^{'}}\right)\right)$$

### 3.2. The Improved Channel Attention Module

**Figure 4:**
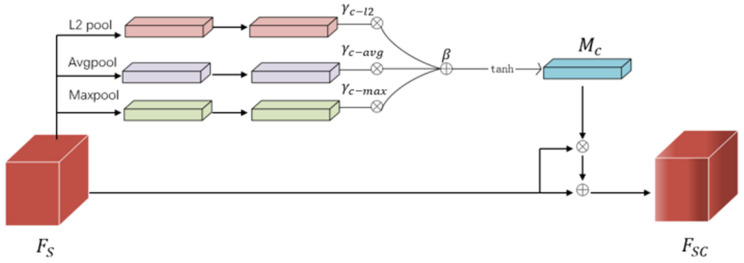
Enhanced channel attention module.

The improved channel attention module is proposed to fuse the multi-level features by considering their different contributions through attention-based weight adjustment. The structure of the enhanced channel attention module is demonstrated in Figure 4. L2Pool operations and cross-channel feature normalization are implemented, and the fully connected layer is replaced by a gating mechanism [35], as shown in Equation (7).

(7) $$L2Pool\left(F_{S}\right)=\frac{\alpha_{c}{\left\{\left[\sum_{i=1}^{H}\sum_{j=1}^{W}{\left(x_{c}^{i,j}\right)}^{2}\right]+\epsilon \right\}}^{\frac{1}{2}}}{{\left(H\times W\right)}^{\frac{1}{2}}}$$

The channel information is aggregated through AvgPool , MaxPool and L2Pool and then can be expressed as $\hat{S_{c-Avg}}$, $\hat{S_{c-Max}}$, and $\hat{S_{c-l2}}$ by normalization. ϵ is a very small constant, which avoids the problem of taking the division at 0, as shown in Equations (8)–(10).

(8) $$\hat{S_{c-l2}}=\frac{L2Pool\left(F_{S}\right)}{{\left\{\left(\sum_{i=1}^{C}{\left[L2Pool\left(F_{S}\right)\right]}^{2}\right)+\epsilon \right\}}^{\frac{1}{2}}}$$

(9) $$\hat{S_{c-Avg}}=\frac{AvgPool\left(F_{S}\right)}{{\left\{\left(\sum_{i=1}^{C}{\left[AvgPool\left(F_{S}\right)\right]}^{2}\right)+\epsilon \right\}}^{\frac{1}{2}}}$$

(10) $$\hat{S_{c-Max}}=\frac{MaxPool\left(F_{S}\right)}{{\left\{\left(\sum_{i=1}^{C}{\left[MaxPool\left(F_{S}\right)\right]}^{2}\right)+\epsilon \right\}}^{\frac{1}{2}}}$$

Appropriate cross-channel interaction can preserve performance while drastically decreasing model complexity. By analyzing the channel attention module in the Squeeze-and-Excitation Network (SENet) [36], Efficient Channel Attention Network (ECANet) [37] empirically shows that avoiding dimensionality reduction is critical for learning channel attention. Therefore, referring to the gating mechanism of the Gated channel transformation (GCT) module [35], the fully connected layer is replaced, and the trainable parameters $\gamma_{c-Avg}$, $\gamma_{c-Max}$, $\gamma_{c-l2}$, and β are designed in this module.

$\gamma_{c-Avg}$, $\gamma_{c-Max}$, and $\gamma_{c-l2}\;$ control the activation state of the three-channel descriptors and assign different weights to the three descriptions, respectively. Finally, the weight map $M_{c}\in R^{C\times 1}$ of channel attention is obtained, as shown in Equation (11).

(11) $$M_{c}=1+tanh\left[\frac{\left(\gamma_{c-Avg}\hat{S_{c-Avg}}+\gamma_{c-Max}\hat{S_{c-Max}}+\gamma_{c-l2}\hat{S_{c-l2}}+\beta \right)}{3}\right]$$

### 3.3. The VA Values Prediction Module

To predict the emotion of the image based on the valence–arousal space, the VA values prediction module is proposed. This section utilizes a residual structure where the input of the network consists of both the global features of the image and the features of the joint emotion map branch.

For the global features of the image, as illustrated in Figure 1, we use the outputs of the last residual block of the pre-trained ResNet101 for each stage. The channel dimensions are denoted as {$c^{2},c^{3},\;c^{4},c^{5}$} for the Conv2, Conv3, Conv4, and Conv5 outputs, respectively. The multi-level feature $F\in R^{\left(c^{2}+c^{3}+c^{4}+c^{5}\right)x^{2}}$ is obtained by a multi-layer feature fusion module, and the global semantic vector $f_{AP}$ is obtained by using the average pooling on F.

For the features of the joint emotion map branch, the union feature $F_{SC}$ is obtained by calculating $F_{S}{\,}^{'}$ with the weight $M_{S}$ of the spatial attention first and then calculating the result with the weight $M_{C}$ of the channel attention. In addition, average pooling is used to obtain the semantic vector $f_{SCAP}$, as shown in Equation (12) and Equation (13) separately.

(12) $$F_{SC}=M_{C}⊗(M_{S}⊙F_{S}{\,}^{'})$$

(13) $$f_{SCAP}=AvgPool\left(F_{SC}\right)$$

As mentioned above, $f_{AP}$ and $f_{SCAP}$ are concatenated to generate the semantic vector f. Finally, f is input into the full connection layers to predict the image emotion based on valence–arousal space.

## 4. Experiment and Results Analysis

### 4.1. Implementation Details

All our experiments are carried out on two NVIDIA RTX 2080Ti GPUs using the PyTorch 1.12.1. Images are resized to 300 × 300. To reduce overfitting, images are randomly horizontally flipped and randomly cropped to 224 × 224 patches as a way of data augmentation. We employed the SGD optimizer to fine-tune all layers with 0.0001 as the learning rate with a 0.0005 weight decay, a 0.9 momentum, and a batch size of 32 for 400 epochs. The valence–arousal labels are normalized to [0, 1], and the dataset is randomly divided into 70% training set, 20% testing set, and 10% validation set. The number of network parameters is 124M, and the average training time is about 20 h.

### 4.2. Datasets

**Figure 5:**
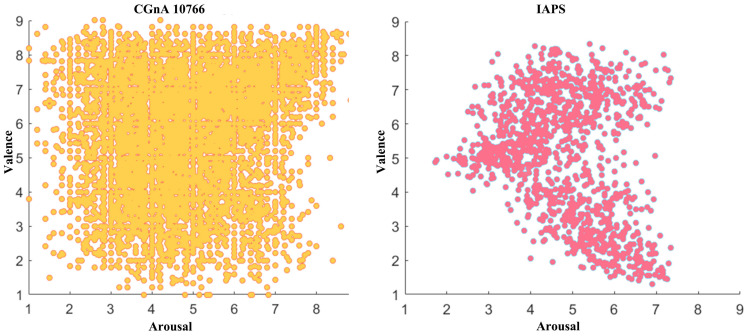
Emotion distribution of CGnA10766 and IAPS based on VA emotion space.

CGnA10766 dataset [13] is composed of 10,766 images searched from Flickr, including eight emotion categories (Amusement, Awe, Contentment, Excitement, Anger, Disgust, Fear, and Sad). The Amazon Mechanical Turk (AMT) assigned the valence–arousal values for all the images in the CGnA10766 dataset ranging from 1 to 9. The valence is ranged from most negative (1 to 3), negative (3 to 5), positive (5 to 7), and most positive (7 to 9). The arousal is ranged from most calm (1 to 3), calm (3 to 5), exciting (5 to 7), and most exciting (7 to 9). Each image has been evaluated by at least five annotators, and the average value has been finally assigned to each image. The distribution of images in the CGnA10766 dataset is shown in Figure 5 [13]. Compared with other image emotion datasets, the emotion distribution of CGnA10766 is more extensive [17].

### 4.3. Performance Comparison

Three commonly used indicators of the regression model are used to evaluate the model performance: Mean Squared Error (MSE), Mean Absolute Error (MAE), and Coefficient of Determination (R^2^) [16]. The proposed network structure ARMNet is compared with other visual emotion regression models, including a pre-training ResNet101, PDANET [16] (we obtained experimental results on the CGnA dataset using this paper’s open-source code), Vision Transformer (ViT) [38], and SCEP [17] with a ResNet101 backbone. The proposed ARMNet performs the best in every indicator. As shown in Table 1, comparing with the best results, experiments on the CGnA10766 dataset show that the performance of ARMNet is improved by 4.74%, 3.53%, 3.62%, 1.93%, 6.29%, and 7.23%, respectively. Experiments show that ARMNet has better robustness and performance by combining the attention mechanism promoted by CBAM with the human eye fixation prediction module.

### 4.4. Ablation Experiments

The proposed ARMNet contains three major components: the multi-level features fusion module, the human eye fixation detection module, and the spatial-channel attention module. To quantitatively show performance improvement, the network structure (the fully connected layers, the holistic feature vector, etc.) is maintained while the major components are removed separately. In addition, the learning rate, weight decay, batch size, and other hyperparameters are the same as in Section 4.1. Finally, MSE is selected as the comparison indicator.

As shown in Table 2, the results reveal the following information:

(1) According to rows 1 and 2 of Table 2, the results of the model with the multi-level features fusion module are better than those without the multi-level features fusion module. The MSE value for valence and arousal of the model with the multi-level features fusion module was reduced by 4.45% and 1.58%, respectively.
(2) According to rows 1, 3, 4, and 5 of Table 2, the eye fixation detection module and the spatial attention mechanism can improve performance. The combination of them performs better than every single module. This proves the necessity of adding a human attention detection module and a spatial attention detection module to the ARMNet. For example, the eye fixation detection module reduced the MSE value for valence and arousal by 1.33% and 1.56%, respectively. Additionally, the spatial attention mechanism module reduces the MSE value by 3.26% in the valence domain, but the MSE value in the arousal domain is almost the same.
(3) According to rows 6 and 7 of Table 2, a comparison shows the performance differences between CAM and SAM. While both combinations (R + M + S + CAM and R + M + S + SAM) yield similar MSE values, R + M + S + SAM slightly outperforms R + M + S + CAM in both the valence and arousal domains. This suggests that although CAM effectively captures channel-wise information, SAM shows more robust performance for spatial attention in the emotional prediction task.
(4) According to rows 6 and 10 of Table 2, the network with the channel attention mechanism module reduces the MSE value for valence and arousal by 2.64% and 1.84% for valence and arousal, respectively, which verifies the validity of the channel attention mechanism module.
(5) When CAM is introduced, as seen in rows 9 and 10 of Table 2, the combination of R + M + S + CBAM + CAM does not outperform R + M + S + SAM + CAM, which delivers better results. This demonstrates that although CBAM has advantages in certain setups, SAM, when combined with CAM, provides more stable and superior performance for emotional prediction.
(6) Furthermore, the spatial-channel attention module is designed based on the CBAM module by adding a gating mechanism, including a spatial attention mechanism module and a channel attention mechanism module. The result is shown in rows 6, 8, 9, and 10 of Table 2. It proves that the CBAM module is effective, while the spatial-channel attention is better. Compared with the CBAM module network, the network with the spatial-channel attention module reduced the MSE value for valence and arousal by 1.03% and 2.49%, respectively.

**Table 2:** Ablation experiments results (“V”and “A” represent “valence” and “arousal”. “R”, “M”, “S”, “SAM”, “CAM”, and “CBAM” represent “ResNet101”, “Multi-level features fusion module”, “Salicon”, “Spatial Attention Mechanism”, “Channel Attention Mechanism”, and “Convolutional Block Attention Module”, respectively. “**↓**” indicates that the value should be as smaller as possible, “**↑**” indicates that the value should be as larger as possible. The bold numbers indicate the best results).

		MSE_V ↓	MSE_A ↓	MAE_V ↓	MAE_A ↓	R^2^_V ↑	R^2^_A ↑
1	R	0.02701	0.02246	0.1289	0.1199	0.3644	0.2467
2	R + M	0.02586	0.02211	0.1259	0.1187	0.3912	0.2586
3	R + S	0.02569	0.02050	0.1259	0.1151	0.3956	0.3126
4	R + SAM	0.02521	0.02082	0.1247	0.1153	0.4067	0.3018
5	R + S + SAM	0.02488	0.02050	0.1237	0.1151	0.4145	0.3124
6	R + M + S + SAM	0.02488	0.02049	0.1235	0.1151	0.4145	0.3129
7	R + M + S + CAM	0.02497	0.02100	0.1239	0.1159	0.4123	0.2955
8	R + M + S + CBAM	0.02449	0.02062	0.1225	0.1150	0.4237	0.3084
9	R + M + S + CBAM + CAM	0.02797	0.02433	0.1304	0.1236	0.3417	0.1839
10	R + M + S + SAM + CAM	**0.02424**	**0.02012**	**0.1217**	**0.1137**	**0.4294**	**0.3249**

### 4.5. Visualization

**Figure 6:**
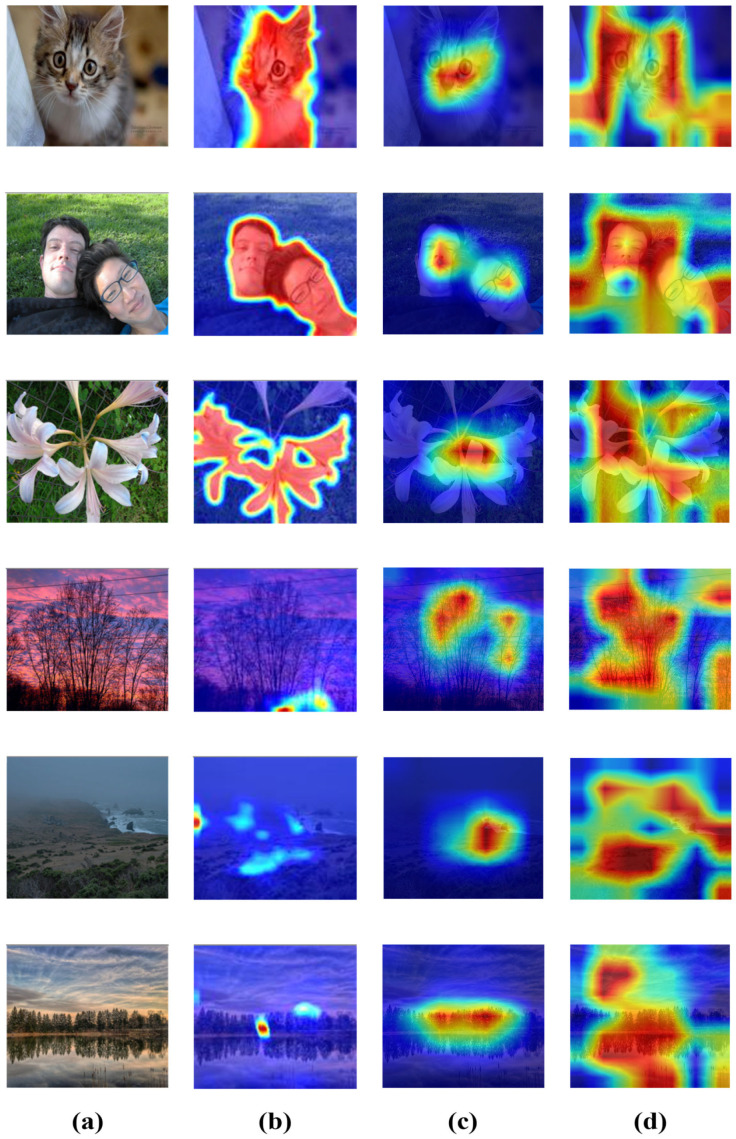
Visualizations of spatial attention: (**a**) the original images; (**b**) the salient object maps; (**c**) the eye fixation maps; (**d**) the union attention region map after integrating the human eye fixation detection module, where the color red represents a higher weight.

Attention map reflects the varying importance of input features and is widely used to improve the interpretability of neural networks. The visualizations of the affective regions and the channel attention of the ARMNet are shown in Figure 6.

#### 4.5.1. Visualization of the Spatial Attention Module

For comparison with existing methods and evaluation of the performance of the union affective region extraction module proposed in this paper, this section conducts a visual analysis. As shown in Figure 6, Figure 6a presents the original picture, while Figure 6b shows one of the affective regions obtained through salient object detection maps using BASNet (Boundary-Aware Salient Object Detection) [39]. Figure 6c,d show the extracted eye fixation map and the union attention region map from ARMNet, respectively.

As shown in Figure 6d, the final attention weights are not only derived from salient objects; this phenomenon is more apparent in images without salient objects. Figure 6c,d show that the eye fixation detection module detects the eye fixation maps, which include the saliency regions, and the spatial attention maps concentrate on the background regions related to emotions. Therefore, the proposed ARMNet can obtain the affective regions with or without the salient objects simultaneously based on the fusion of the human eye fixation detection module and spatial attention mechanism. In other words, the union affective region extraction module enhances the model’s ability to capture emotional information.

**Figure 7:**
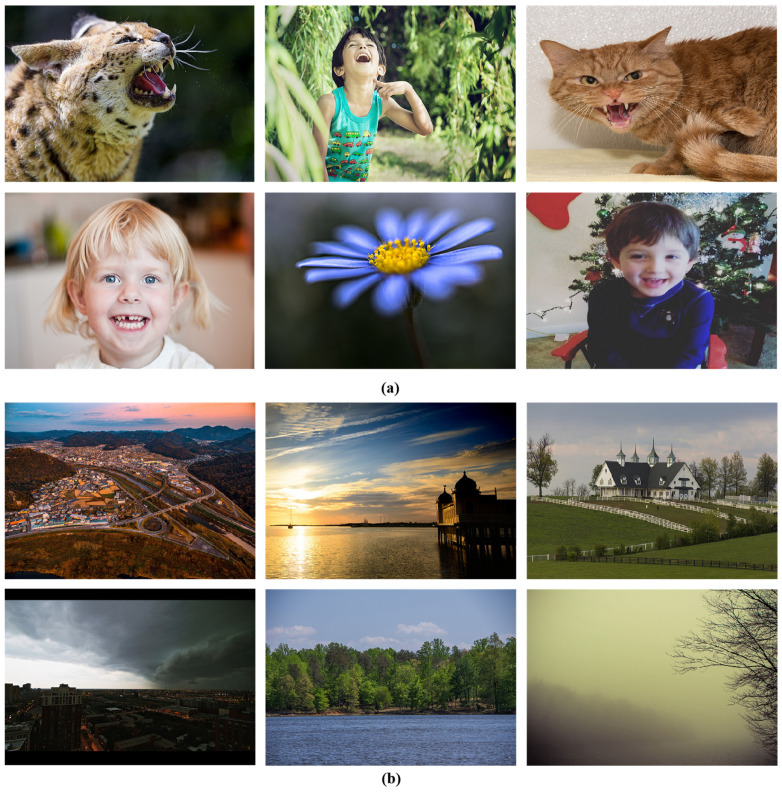
Images from CGnA10766 dataset: (**a**) images with specific target objects (person, animal, etc.); (**b**) images without a specific target subject.

To further illustrate the effectiveness of the proposed fusion attention mechanism, the images of the CGnA10766 dataset were divided into two categories, as shown in Figure 7. The first category consists of images that contain a specific target object, such as people or animals. The second category includes images without a specific target object, such as landscapes. The first and second categories of images are 6287 and 4479, respectively. Moreover, as shown in Table 3, the valence prediction error of the first category is much smaller than that of the second category, whereas the arousal prediction errors for both categories are almost the same.

#### 4.5.2. Visualization of the Improved Channel Attention

**Figure 8:**
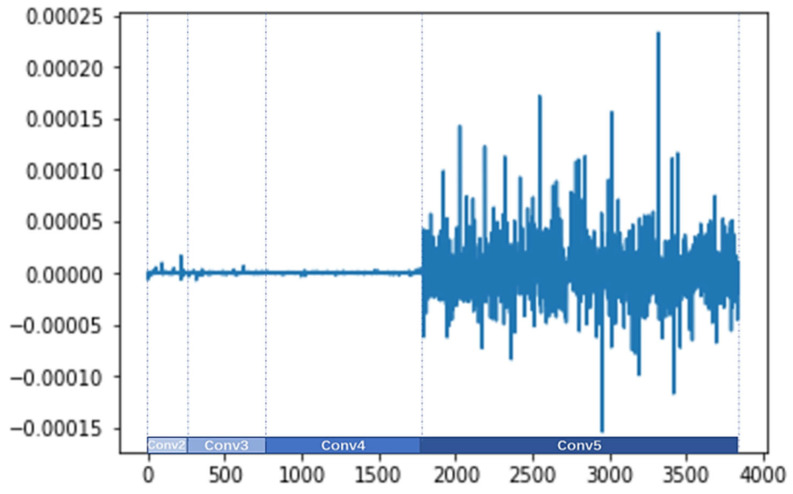
Visualization of $\gamma_{c-Avg}$.

**Figure 9:**
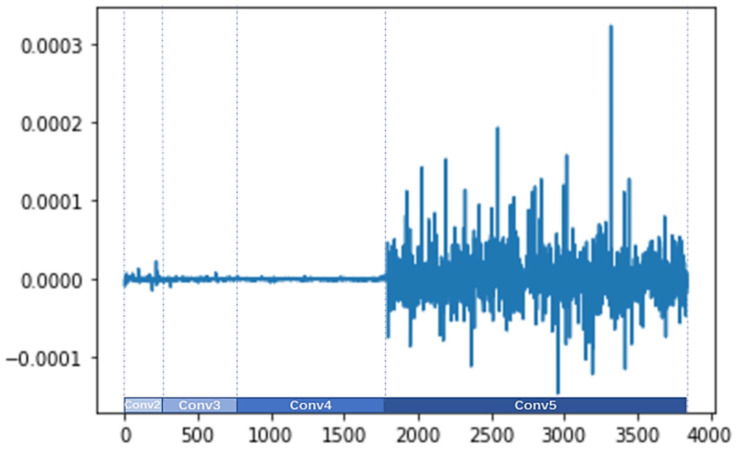
Visualization of $\gamma_{c-Max}$.

**Figure 10:**
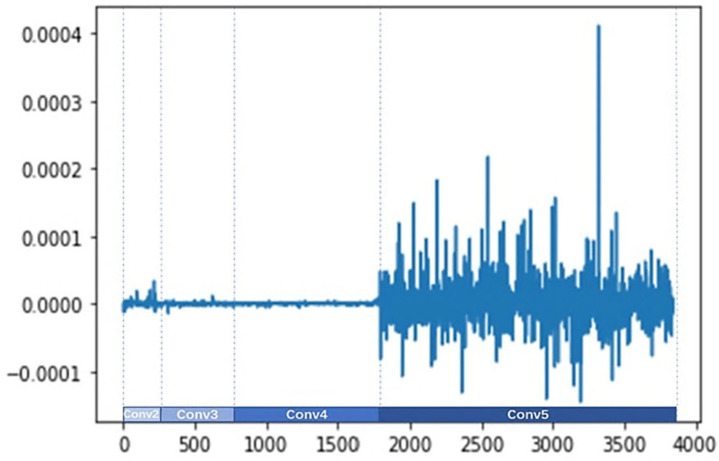
Visualization of $\gamma_{c-l2}$.

The three weight parameters of the improved attention channel $\gamma_{c-Avg}$, $\gamma_{c-Max}$, and $\gamma_{c-l2}$ are obtained by Equation (11), and the dimension of the channel is 3680. The values of $\gamma_{c-Avg}$, $\gamma_{c-Max}$, and $\gamma_{c-l2}$ are shown in Figure 8, Figure 9, and Figure 10, respectively. The visualization results reveal the following information:

(1) The different parameters $\gamma_{c-Avg}$, $\gamma_{c-Max}$, and $\gamma_{c-l2}$ indicate that the network assigns different importance to different channel feature descriptors.
(2) The specific values of $\gamma_{c-Avg}$, $\gamma_{c-Max}$, and $\gamma_{c-l2}$ are different. The gating weights of the output features from the Conv3 and Conv4 branches are approximately zero, and the gating weights of the output features from the Conv2 branch are small. However, the gating weights of the output features from the Conv5 branch are bigger and fluctuate more sharply, which show a greater influence on the final prediction.

## 5. Conclusions

Emotion recognition and processing are essential research directions for developing new intelligent sensing systems in the future. Complex emotions can be described better based on dimensional emotion space, including the subtle emotion representation. This precise representation of emotion necessitates the use of dimensional emotion prediction methods to capture emotional information in images with maximum accuracy and comprehensiveness. However, existing methods mainly extract affective regions with salient objects, neglecting the joint influence of objects and background for the emotion of the image. And even with the same object, there may be differences in emotion due to different backgrounds. Additionally, they fused multi-level features without considering their different contributions to emotion prediction, which makes it challenging to discriminate between valuable and useless features.

This paper proposes an image emotion prediction network named ARMNet. In ARMNet, a union affective region extraction method that combines eye fixation detection and attention detection is proposed to represent the joint influence of objects and background. And the multi-level features are fused with considering their different contributions by an improved channel attention mechanism. We compare the proposed network with other advanced methods on the CGnA10766 dataset. The proposed network performs the best in every indicator. The performance of valence and arousal, as measured by MSE, MAE, and R², has improved by 4.74%, 3.53%, 3.62%, 1.93%, 6.29%, and 7.23%, respectively. The effectiveness of each module is also proved by ablation experiments.

## Figures and Tables

**Table 1:** Comparison experiment results (“V” and “A” represent “valence” and “arousal”, respectively. “**↓**” indicates that the value should be as smaller as possible, “**↑**” indicates that the value should be as larger as possible. The bold numbers indicate the best results).

	MSE_V ↓	MSE_A ↓	MAE_V ↓	MAE_A ↓	R^2^_V ↑	R^2^_A ↑
ResNet101 [32]	0.02701	0.02246	0.1289	0.1199	0.3644	0.2467
PDANet [16]	0.02589	0.02083	0.1263	0.1159	0.3909	0.3014
ViT [38]	0.03462	0.02705	0.1455	0.1351	0.1852	0.0927
SCEP [17]	0.02539	0.02117	0.1261	0.1160	0.4024	0.2900
ARMNet (ours)	**0.02424**	**0.02012**	**0.1217**	**0.1137**	**0.4294**	**0.3249**

**Table 3:** Experimental results on two categories of images with or without specific target subjects: (a) images with specific target objects (person, animal, etc.); (b) images without a specific target subject. “**↓**” indicates that the value should be as smaller as possible, “**↑**” indicates that the value should be as larger as possible.

	MSE_V ↓	MSE_A ↓	MAE_V ↓	MAE_A ↓	R^2^_V ↑	R^2^_A ↑
(a)	0.02528	0.02134	0.1258	0.1167	0.3456	0.2509
(b)	0.01696	0.02167	0.1012	0.1172	0.5229	0.2858

## Data Availability

The datasets used in this paper are openly available at: https://figshare.com/articles/CGnA10766_Dataset/5383105 (accessed on 1 September 2020).
